# Increasing Maternal Vaccination Awareness, by Working With Women Influencers in Kawempe Division, Uganda: A Brief Report

**DOI:** 10.1097/INF.0000000000004635

**Published:** 2025-02-14

**Authors:** Mary Kyohere, Phiona Nalubega, Hassan Ssemere, Agnes Ssali, Kirsty Le Doare

**Affiliations:** From the * Makerere University-Johns Hopkins University Research Collaboration, Kampala, Uganda; † City St George’s University of London, School of Health & Medical Sciences, Institute for Infection and Immunity, London, United Kingdom; ‡ MRC/UVRI and LSHTM Uganda Research Unit, Entebbe, Uganda.

**Keywords:** vaccination awareness, maternal vaccination, public engagement

## Abstract

Although the World Health Organization recommends 2 tetanus vaccine-containing injections in pregnancy, in Uganda, only 59.2% of pregnant women received a 2nd dose in 2022. We set out to (1) create awareness of maternal vaccine-preventable diseases, maternal immunization benefits and vaccination safety through the use of media; (2) determine the effectiveness of maternal immunization campaigns and (3) assess stakeholder’s acceptability of project interventions’ delivery methods.

The World Health Organization (WHO) recommends 2 doses of tetanus toxoid-containing injections during any pregnancy to realize full protection, unless full immunity has been achieved from previous vaccinations.^1^ However, WHO data in 2022 indicated that only 59.2% of pregnant women and women of childbearing age in Uganda received a 2nd injection of tetanus toxoid,^2^ indicating ineffective coverage and compromised protection for women and their newborns. A previous qualitative study done by our team on public engagement showed widespread misconceptions on vaccination. This previous study explored understanding of maternal vaccination and potential barriers to delivery and uptake of vaccination in pregnancy: in focus group discussions with health workers, pregnant women as well as in-depth interviews with pregnant women who have experience with maternal vaccination, it was found that women had limited knowledge of vaccines and had concerns over the effectiveness and safety of vaccines for their unborn babies. The women also indicated that husbands, peers, mothers and mothers-in-law are key influencers to uptake of maternal immunization.^3^

The purposes of this study are as follows: the project, carried out by Makerere University-Johns Hopkins University (MU-JHU) Research Collaboration, therefore set out to (1) create awareness of maternal vaccination benefits and vaccination safety among people who are key influencers to uptake of maternal vaccination, (2) determine the effectiveness of a maternal vaccination campaign and ascertain whether there is increased awareness and (3) assess stakeholder’s acceptability of project interventions’ delivery methods and effectiveness of training on community influencers.

## MATERIALS AND METHODS

With the help of the community advisory board chairperson at MU-JHU and independent interviews with community members, key influential people in the target communities were named, and we identified 20 community influencers from 10 parishes surrounding Kawempe National Referral Hospital, a major maternal-child health facility where several of MU-JHU’s research projects are carried out. With the help of the community advisory board chairperson and the hospital in charge of the antenatal clinic, the 10 parishes were purposely selected among those closest to the hospital. Two influencers were selected from each of the 10 parishes and included religious leaders, village health team members, businesspeople and local council leaders. Both women and men were involved. The influencers were invited to meetings to discuss maternal vaccination. As members of the target community, we received their views and answered their questions about maternal vaccination. We then trained them on maternal vaccination using the Uganda Ministry of Health (MoH) Vaccination handbook, to ensure the message disseminated was in keeping with the MoH message. Following the training, the community influencers went on a door-to-door campaign that lasted 1 month, interacting with community members at their homes and other locations outside the hospital, to raise awareness about maternal vaccination. The community influencers had feedback diaries (Table, Supplemental Digital Content 1, http://links.lww.com/INF/F836) where they documented the number of people interacted with, and would return these to the team weekly.

While most influencers were able to dedicate time to the door-to-door campaign, their involvement was balanced with their regular responsibilities. Village health teams and local council leaders were accustomed to mobilizing communities for health programs and were flexible in their schedules. None of the influencers reported being engaged in demanding businesses that would significantly hinder their community engagement.

The community influencers helped cocreate a short film and audio messages on maternal vaccination. The film script was reviewed and approved by a team from the MoH. The use of film and audio messaging, specifically for maternal vaccination had not previously been done in Uganda. The media was in English and the local language, using local well-recognized personnel to do the acting, and set in a recognizable environment. The film promoted male involvement in maternal vaccination, depicting dialogue between a husband and wife, with the husband eventually escorting the wife to the hospital and having his questions answered by medical personnel. The audio messages were conversations between friends, discussing the benefits of maternal vaccination, addressing concerns and advising that maternal vaccine is beneficial to both mother and child. The short film was shown in antenatal waiting areas, whereas the audio messages were broadcast as radio spot messages over 3 stations local to the target community, in both English (KFM) and the dominant local language, Luganda (Radio Bilal and Bukedde Radio). The radio spot messages were broadcast for a month, during which the door-to-door campaign and community engagement sessions detailed below were running concurrently. The community influencers asked the community members they met during the door-to-door campaign if they had heard the message being broadcast over the radio to assess how far the message was reaching.

Two of the community influencers were hosted at a local radio station during a health talk program, for a more in-depth discussion following the radio spot messages. Members of the public were able to call in and ask questions which the influencers responded to. The project had planned for only one such radio program, but based on the positive reception, the team was called back for 2 other sessions.

With the help of the community influencers, we organized 10 community engagement sessions, whereby members of the community gathered with the project team members. During these sessions, the film was shown, and members of the community interacted with project staff and community influencers who responded to their questions about maternal vaccination. At each of these community engagement sessions, pre- and postfilm question and answer sessions were held, to assess existing knowledge and learning among the community members in attendance. These were free-style discussions with the project staff asking participants what they knew before the film, and comparing it with what they knew after the film, and this would be reported in the session report. Please see Table, Supplemental Digital Content 2, http://links.lww.com/INF/F837, that summarizes the project activities.

Finding the people in the communities to share the information with them as opposed to waiting for them to come to the facility was also a different approach to information dissemination.

As part of the project, we developed and distributed t-shirts portraying the message: “Maternal Vaccines are Safe, Effective and Free.” These were worn by the community influencers during the door-to-door campaign and distributed to the attendees at the community engagement sessions, to further carry the message of maternal vaccines into the community.

The film and audio script and t-shirt messaging were all reviewed and endorsed by the Uganda MoH. Permission to carry out the community engagement activities was sought and received from the local council and health officials but no consent was obtained from individual attendees since it was not a study.

## RESULTS

The door-to-door campaign reached more than 900 community members of various ages and included husbands, parents and in-laws of pregnant and nonpregnant women of childbearing age, as well as the women themselves. The 10 community engagement sessions involved more than 430 community members.

Feedback received during the campaign, documented in Influencer Feedback Diaries and the community engagement sessions (see questions, Supplemental Digital Content 3, http://links.lww.com/INF/F838), showed that the radio spot and short film messages were well received; they acknowledged the film as a plausible way of creating awareness for maternal vaccines since most women like watching films, and they said films communicate better than other information, education and communication materials.

Sixty-three percent of community members that the community influencers interacted with had heard the radio broadcasts and were able to quote the benefits of maternal vaccination. By the end of the community engagement sessions, there was increased knowledge about maternal vaccines displayed by the attendees, with participants acknowledging that they had learnt about the maternal vaccines, benefits of vaccinating and dangers of not vaccinating.

Before the film viewing and discussions, most participants had limited knowledge about maternal vaccines since they could not mention the Tetanus-Diphtheria vaccine as the only vaccine offered to pregnant women in Uganda at the time. Regarding the perceptions of maternal vaccines, most concerns were about vaccine safety such as community myths and misconceptions about vaccines, fear of pain and fear of side effects. After the film viewing, the participants expressed better understanding of the importance of the maternal vaccines, the safety profile and the required scheduling, and dangers of not vaccinating.

Members of the community appreciated the male involvement among the influencers, and in the campaign and community engagement sessions as pregnancy and maternal health are widely viewed as women’s issues. Women mentioned that their husbands “were never engaged in any aspect of maternal health including HIV testing since they weren’t interested,” “… thought that it wasn’t their role to participate in health care of their children or wives”; “that was a role meant for women only and theirs was only to work and provide food at home,”; so they were happy to see men involved in the community engagement and campaigns.

The distribution of branded t-shirts meant the message was constantly visible in the community. A copy of the video message was given to the main health facility, to be shown in the antenatal and other waiting areas. The audio and video messages were developed with the Uganda MoH communications team, ensuring a wider reach. The messages are also available on the IMPRINT website^4,5^ and can be used by other teams seeking to do something similar. Future plans include translation of the media to other local languages to reach audiences outside Central Uganda, as well as extended door-to-door campaigns, community engagement and radio campaigns lasting longer than a month. For future studies, we would want to assess whether the level of uptake of maternal vaccination in this community has increased, or is higher than in other parts of the country that might not have received the same messaging.

### Limitations

This public engagement program was conducted during the coronavirus disease 2019 pandemic period in early 2021, while aspects of social distancing were still in place. As a result, the movements of the community influencers and the number of people that could be gathered at a given time were limited. This was a community engagement program, not a formal study so formal data collection and analysis was not done; therefore, the extent of effectiveness of the project activities is not fully known. The community engaged was an urban community; therefore, the results might differ in a rural community.

## CONCLUSION

The use of audio and video messaging is an acceptable and well-received method of educating the public on maternal vaccination. Radio messages can be used to reach a large percentage of the population. The involvement of men in health campaigns is appreciated and can potentially help improve the uptake of maternal vaccination.

## ACKNOWLEDGMENTS

*The authors thank the community in the 10 parishes of Kawempe Division, Kampala, Uganda, for hosting the project teams; Radio Bilal, KFM Radio and Bukedde Radio for airing the radio spot messages and the latter for hosting the team on several talk shows; Nes Motion Media for directing and producing the video and audio messages; and Uganda Ministry of Health and local authorities for their input and support*.

## Supplementary Material



## References

[1] World Health Organization. Protecting All Against Tetanus: Guide to Sustaining Maternal and Neonatal Tetanus Elimination (MNTE) and Broadening Tetanus Protection for all Populations. Immunization, Vaccines and Biologicals (IVB); 2019. ISBN: 978 92 4 151561 0

[2] World Health Organization, Geneva, Switzerland. Protection at birth (PAB) against neonatal tetanus vaccination coverage and vaccination coverage among pregnant women of Tetanus toxoid-containing vaccine (TT2+/Td2+) and Pertussis-containing vaccine – Uganda. Available at: https://immunizationdata.who.int/global?topic=Vaccination-coverage&location=UGA. Accessed September 26, 2024.

[3] Nalubega P Karafillakis E Atuhaire L. Maternal vaccination in Uganda: exploring pregnant women, community leaders and healthcare workers’ perceptions. Vaccines. 2021;9:552. 34070536 10.3390/vaccines9060552 PMC8230088

[4] LINQ management, short film on maternal vaccination. 2021. Available at: https://www.youtube.com/watch?v=mUD0DJuodxk. Accessed September 26, 2024.

[5] Radio spot message in Luganda and English. Available at: intranet.imprint-network.co.uk/wp-content/uploads/2021/12/IMPRINT_radio_message_on_tetanus_vaccination.mp3. Accessed September 26, 2024.

