# Supplementary material for: Increasing Maternal Vaccination Awareness, by Working With Women Influencers in Kawempe Division, Uganda: A Brief Report
Source: Pediatr Infect Dis J. 2025 Feb 14;44(2):S146–8. doi: 10.1097/INF.0000000000004635 (PMC12178167; doi:10.1097/INF.0000000000004635)
Supplement: Supplementary file 1 [file inf-44-s146-s001.pdf]

Supplemental Digital Content 1. Influencer Feedback Diary

IMPRINT Feedback Diary

Date: ____

1: Details of person talked to:

a. Age bracket:

____ ≤ 20

____ 21 – 30

____ 31 – 40

____ 41 – 50

____ 51 – 60

b. Category:

____ Non-pregnant woman

____ Pregnant woman

____ Husband

____ Parent of pregnant woman (select whether _____Father or _____Mother)

____ In-law of pregnant woman

2: Feedback:

a. Have they heard the radio spot message on vaccination?

Yes ______ No _______

If yes, what 3 key messages have they picked from the radio sport message?

____

____

____

Do they have any questions from the radio spot message?

____

____

____

IMPRINT Feedback Diary Page 1 of 2

IMPRINT Feedback Diary

Go through the section on Maternal Vaccinations from the handbook with the person.

b. What 3 key messages have they picked from the handbook message?

____

____

____

Do they have any questions from the handbook message?

____

____

____

3: Are there any other comments?

____

____

____

IMPRINT Feedback Diary Page 2 of 2
