# Supplementary material for: Increasing Maternal Vaccination Awareness, by Working With Women Influencers in Kawempe Division, Uganda: A Brief Report
Source: Pediatr Infect Dis J. 2025 Feb 14;44(2):S146–8. doi: 10.1097/INF.0000000000004635 (PMC12178167; doi:10.1097/INF.0000000000004635)
Supplement: Supplementary file 2 [file inf-44-s146-s002.pdf]

SUPPLEMENTAL DIGITAL CONTENT 3. Feedback guiding questions:

Pre-Assessment questions

1. What are some of your perceptions about maternal vaccines?
2. Would you recommend community members to take maternal vaccinations?
3. What are some of the challenges or gaps you see in provision of maternal vaccines?

Post-Assessment

1. What do you think about our film?
2. What aspect of the film touched you the most and why? (**Emotional engagement**)
3. Do you have any recommendations?
